# Cichoric acid improves isoproterenol‐induced myocardial fibrosis via inhibition of HK1/NLRP3 inflammasome‐mediated signaling pathways by reducing oxidative stress, inflammation, and apoptosis

**DOI:** 10.1002/fsn3.3758

**Published:** 2023-10-12

**Authors:** Xizhen Cheng, Yuling Zhang, Haochuan Guo, Xinnong Li, Yanan Wang, Yongxing Song, Hongfang Wang, Donglai Ma

**Affiliations:** ^1^ School of Pharmacy Hebei University of Chinese Medicine Shijiazhuang China; ^2^ Traditional Chinese Medicine Processing Technology Innovation Center of Hebei Province Shijiazhuang China; ^3^ Hebei Technology Innovation Center of TCM Formula Preparations Shijiazhuang China

**Keywords:** cichoric acid, HK1/NLRP3, myocardial fibrosis, signaling pathway

## Abstract

Cichoric acid (CA), a natural phenolic compound found in many plants, has been reported to have antioxidant, anti‐inflammatory, hypoglycemic, and other effects. The aim of this study was to determine the potential role and underlying mechanisms of CA in isoproterenol (ISO)‐induced myocardial fibrosis (MF). The MF model was induced by subcutaneous ISO injection in mice. Blood and heart tissue were collected for examination. Hematoxylin and eosin staining and Masson's trichrome staining were used to evaluate the histopathological changes and collagen deposition. The production of reactive oxygen species markers was observed by fluorescence microscopy, the degree of cardiomyocyte microstructure injury was observed by transmission electron microscope, and oxidative stress factors were detected by kit method, and the effect of CA on inflammatory factors was detected by ELISA. The expression levels of collagen proteins and signaling pathways were further investigated by western blotting. The results showed that CA inhibited the expression of ISO‐induced proinflammatory factors (TNF‐α, IL‐1β, and IL‐18) and proteins (HK1, NLRP3, caspase‐1, cleaved‐caspase‐1, and ASC), and regulated the expression of apoptotic factors (caspase‐3, cleaved‐caspase‐3, Bax, and Bcl‐2). The results indicated that CA may regulate the HK1/NLRP3 inflammasome pathway by inhibiting HK1 expression and play a protective role in MF.

## Graphical abstract

In this work, we confirmed that chicoric acid inhibits isoproterenol‐induced myocardial fibrosis by regulating the HK1/NLRP3 inflammasome pathway, thereby inhibiting inflammation, oxidative stress, and apoptosis. Based on these findings, chicoric acid may be a promising drug for future clinical applications.

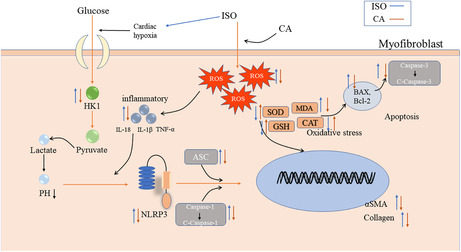

## 1 INTRODUCTION

Myocardial fibrosis (MF) is closely related to a variety of cardiovascular diseases and is caused by various cardiac injuries, such as myocardial infarction and hypertension (Zhang et al., 2021; Zhou et al., 2016). Fibrosis is not a disease, but the result of a tissue repair response. The degree of MF is generally considered to predict adverse outcomes. The adult heart had little regenerative capacity and can only heal by forming scars, and reparative MF is primarily a process in which dead heart muscle cells were replaced by collagen‐based scars (Tallquist, 2020). When tissue is damaged, components in the extracellular matrix (ECM) are synthesized and degraded out of balance, which is the necessary stage for tissue repair (Venkatachalam et al., 2009). Cardiac fibroblasts are the main stroma‐producing cells that underwent ECM deposition through pathological stimulation in fibrosis. The initial ECM deposition is to maintain the integrity of the heart muscle tissue, but persistent fibrosis can lead to heart failure (Frangogiannis, 2021). When beta‐adrenergic receptors are continuously activated, the synthesis and secretion of type I and type III collagen are continuously increased, leading to pathological MF. It reflects the repair of fibrotic response of cardiomyocyte necrosis, the release of fibroblast mediators by stimulated cardiomyocytes, or the activation of immune cells (Frangogiannis, 2021). Isoproterenol (ISO) is a synthetic β‐adrenergic receptor agonist that has been widely used to induce models of cardiac fibrosis (Sun et al., 2021).

Long‐term chronic inflammation can lead to repair MF. Endogenous signals of aseptic inflammation are mediated by inflammasome (Sun et al., 2021). Nucleotide‐bound oligomeric domain‐like receptor protein‐3 inflammasome (NLRP3 inflammasome) is a oligomeric protein complex consisting of NLRP3, active caspase‐1, and apoptosis‐associated speck protein (ASC) that has been shown to induce inflammation and fibrosis by regulating the maturation of IL‐1β and IL‐18 (You et al., 2022). Previous studies have demonstrated that NLRP3 activation contributes to renal fibrosis (You et al., 2022), hepatic fibrosis (Niu et al., 2022; Yuan et al., 2022), and pulmonary fibrosis (Joshi et al., 2022), and inhibition of NLRP3 activation reduces the degree of fibrosis. The specific mechanism of NLRP3 on MF has not been reported in current studies. Studies have shown that inflammasome activation initially occurs in cardiac fibroblasts (Prabhu & Frangogiannis, 2016). Ranheim's study found that NLRP3 was upregulated in the myocardium after myocardial infarction, mainly in fibroblasts (Sandanger et al., 2013).

Normally, the heart's ATP comes mainly from fatty acid oxidation. If myocardial tissue is severely ischemic or without a blood supply, then oxidative phosphorylation of glucose is inhibited, and ATP produced by glycolysis becomes the only energy source for cardiomyocytes (Tran & Wang, 2019). Changes in the glucose and lipid metabolism in the heart have been shown to trigger cardiomyocyte apoptosis, myocardial hypertrophy, and heart fibrosis (Li et al., 2023). Simply increasing glycolysis may cause the heart to produce lactate and protons (Jaswal et al., 2011). Excessive production of lactic acid induced proliferation and differentiation of myofibroblasts in a pH and TGF‐β1‐dependent manner and exacerbated cardiac fibrosis. Hexokinase (HK) has four different isoenzymes, including HK1 and HK2 (Ji et al., 2022). During glycolysis, HK catalyzed the conversion of glucose to glucose‐6‐phosphate (Jin et al., 2021). HK1, an isoenzyme of HK, is the initial rate‐limiting enzyme for glucose phosphorylation. HK1 promoted inflammatory development by accelerating glycolysis and is a key factor in NLRP3 inflammasome activation and pyroptosis in many diseases (Chen et al., 2023). Jin et al., 2021 found that the expression of HK1 increased under the stimulation of oxidized low‐density lipoproteins, and the overexpression of HK1 aggravated the activation of the glycolytic capacity and the formation of the NLRP3 inflammasomes. However, the effect of NLRP3 regulation by HK1 on MF has been less studied. Therefore, this study was designed to protect against ISO‐induced MF by targeting HK1 to regulate NLRP3 inflammasome activation and inhibit caspase‐1 and IL‐1β.

**FIGURE 1:**
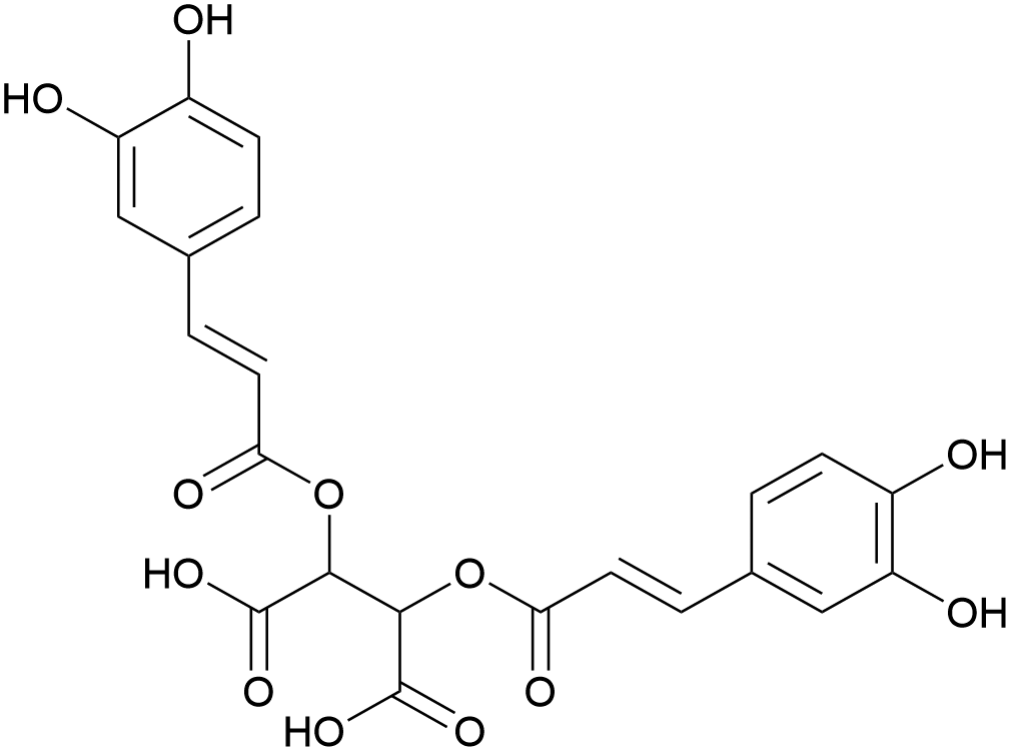
Chemical structure of cichoric acid.

Cichoric acid (CA; Figure 1) is a natural phenolic compound found in many plants, such as chicory, echinacea, and dandelion, and has been reported to show antioxidant, anti‐inflammatory, antihyperglycemic activities, and other effects (Ding et al., 2020; Mohamed Sharif et al., 2021; Xue et al., 2017). Chicory and dandelion are used in daily life not only as food, but also as medicinal materials in clinical and traditional medicine (Ding et al., 2019). CA has been reported to inhibit lipopolysaccharide‐ATP‐induced NLRP3 activation and caspase‐1 activation in HT‐29 cells and to inhibit NLRP3 inflammasome‐mediated secretion of IL‐1B and IL‐18 (Ding et al., 2019). CA stimulated phagocytes through antioxidant activity, and it has been found that CA can regulate the production of intracellular reactive oxygen species (ROS), leading to the activation of PI3K/Akt and MAPK signaling pathways for mitochondria‐dependent apoptosis of 3T3‐L1 preadipocytes (Xiao et al., 2013). Studies have found that the excessive production of ROS is thought to play a key role in pathologies, such as ischemia–reperfusion or neurodegenerative diseases, as well as in various forms of stimulation‐induced apoptosis (Chernyak et al., 2006). CA also effectively prevents monosodium urate (MSU) crystal‐induced macrophage inflammatory response by inhibiting the NF‐κB signaling pathway (Qu et al., 2022). CA also reduced streptozotocin‐induced hyperglycemia in mice by regulating pancreatic cell apoptosis (Ikushima et al., 2022). However, the study of CA in MF remains to be addressed.

This study hypothesized the protective effect of CA on ISO‐induced MF in Kunming mice. The protective effect of CA on the heart was also discussed from the aspects of oxidative stress, inflammation, and apoptosis, and its potential mechanism is closely related to the regulation of the anti‐inflammatory effect of HK1‐NLRP3 signaling pathway.

## 2 MATERIALS AND METHODS

### 2.1 Animals

Fifty Kunming male mice (weighing 18–22 g) were obtained from Liaoning Changsheng Biotechnology Co., Ltd. The mice were adaptively fed for a week at an ambient temperature of 23 ± 2°C and humidity of 50%–60%. The experimental procedures and protocols were approved by the Committee on the Ethical Use of Animals of Hebei University of Chinese Medicine (DWLL2021106).

### 2.2 Drugs

CA was purchased from Shanghai Shifeng Biological Technology Co., Ltd. It was prepared as a suspension by dissolving it in 0.5% sodium dimethyl sulfoxide (DMSO) and shaking vigorously before use. ISO was obtained from Cayman Chemical.

### 2.3 Experimental design

Fifty Kunming mice were randomly divided into five groups (*n* = 10): the blank group (Cont group), model group (ISO group), CA low‐dose group (CA‐L group), CA high‐dose group (CA‐H group), and CA‐alone administration group (CA group). The mice MF model was induced by subcutaneous injection of ISO 10 mg/kg/day (Xue et al., 2021) for 14 days. CA was dissolved in 0.9% normal saline (Shijiazhuang No. 4 Pharmaceutical Co., Ltd.). At the same time, the CA‐L group, CA‐H group, and CA group were treated with CA (Zhu et al., 2017) at 10, 20, and 20 mg/kg, respectively. Cont group was given intragastric administration and subcutaneous injection of 0.9% normal saline (10 mg/kg/day). The experiment lasted for 14 days. After 14 days, the mice were anesthetized by intraperitoneal injection of sodium pentobarbital solution (50 mg/kg), blood was taken, heart was taken, and set aside.

### 2.4 Histological analysis

The mouse hearts were taken out and fixed in 4% paraformaldehyde solution, then the heart tissues were gradient eluted with ethanol, embedded in paraffin, after cutting into 5 μm paraffin slices. The sections were stained with hematoxylin eosin (H&E) and Masson's trichrome. Finally, the slices were observed and photographed under a light microscope.

### 2.5 Serum biochemical analysis

After orbital blood was taken from mice, it was centrifuged at 1360 *g* for 10 min and measured according to the relevant guidelines of the reagent supplier. Total serum was tested for creatine kinase (CK‐MB), lactate dehydrogenase (LDH; Jian Cheng, Nanjing, China; Catalog: E006‐1‐1, A020‐1‐2), and cardiac troponin I (cTnI; Hua Ying Biological Engineering Institute, Beijing, China; Catalog: HY‐D0022).

### 2.6 Antioxidant activity assay

We used MDA kit (Catalog: A003‐1), SOD kit (Catalog: A001‐1), CAT kit (Catalog: A007‐2), and GSH kit (Catalog: A006‐2) to evaluate the oxidative stress levels. All kits were purchased from Jian Cheng Biological Engineering Institute (Nanjing, China).

### 2.7 Electron microscopy

The mouse hearts were fixed in 2.5% glutaraldehyde solution, rinsed with sodium carbonate solution, then fixed in 1% osmium tetroxide, dehydrated with acetone, embedded, and sliced. Finally, stained with uranyl acetate and lead citrate and Tecnai G2 Spirit transmission electron microscope (TEM; ZOOM‐1 HC‐1, Hitachi, Japan) observed and analyzed the results.

### 2.8 ROS detection

The heart tissue was frozen into sections using a cryosecting mechanism, stained with ethidium dihydroin in a dark incubator for 30 min, and then stained with DAPI solution at room temperature for 10 min. The images were observed and collected under a fluorescence microscope. The abovementioned reagents are provided by Wuhan Servicebio Company.

### 2.9 Enzyme‐linked immunosorbent assay (ELISA)

We followed the instructions of the ELISA kit (Servicebio) to detect the levels of TNF‐α (Catalog: 88–7324), IL‐18 (Catalog: EK218‐03), and IL‐1β (Catalog: SP12667) in serum.

### 2.10 Western blot analysis

The myocardial tissue was disrupted into a tissue homogenate by RIPA lysate buffer and a tissue fragmentation instrument, and the supernatant was collected after centrifuging at 15984 *g* for 10 min. The protein tissue homogenate with an appropriate quantity of protein was added to an appropriate volume of loading buffer. Protein samples were boiled for 10 min before loading, and then stored at −80°C after cooling. The target protein was separated by electrophoresis. The membrane was incubated in the membrane transfer solution for 1–2 h for membrane transfer. Then the membrane was incubated for 2 h with 5% defatted milk powder prepared with Tris‐buffered saline with 0.1% Tween 20 to block the polyvinylidene fluoride membrane. Dilute the antibody with a concentration of 1:1000 of anti‐α‐SMA (Servicebio, Catalog: GB111364), anti‐collagen I (Servicebio, Catalog: GB11022), anti‐collagen III (Servicebio, Catalog: GB11023), anti‐Bax (Servicebio, Catalog: GB12690), anti‐Bcl‐2 (Servicebio, Catalog: GB113375), anti‐caspase‐3 (Servicebio, Catalog: GB11767C), anti‐c‐caspase‐3 (Servicebio, Catalog: GB11767C), anti‐HK1 (Beyotime, Catalog: AF1726), anti‐NLRP3 (Affinity, Catalog: DF7502), anti‐ASC (BIOSS, Catalog: BS‐6741R), anti‐c‐caspase‐1 (CST, Catalog: 98332), and anti‐caspase‐1 (Servicebio, Catalog: GB11383). Anti‐GAPDH (Servicebio, Catalog: GB15002, dilution 1:2000) was used as the internal standard. The next day, the membranes were incubated at room temperature for 1 h in the dark with secondary antibodies (goat anti‐mouse IgG, Catalog: GB25301; or goat anti‐rabbit IgG, Servicebio, Catalog: GB23303; both from Servicebio, dilution 1:5000). The bands were scanned and the relative expression of the target proteins was calculated.

### 2.11 Statistical analysis

The experimental data were expressed as mean ± standard deviation (SD). The Student's *t* test analyzed the significant difference between the two groups. One‐way analysis of variance (ANOVA) analyzed significant differences and then performed post hoc Tukey's test in three or more groups. The *p* < .05 was considered statistically significant.

## 3 RESULTS

### 3.1 Effects of CA on ISO‐induced histopathological changes

#### 3.1.1 H&E staining

**FIGURE 2:**
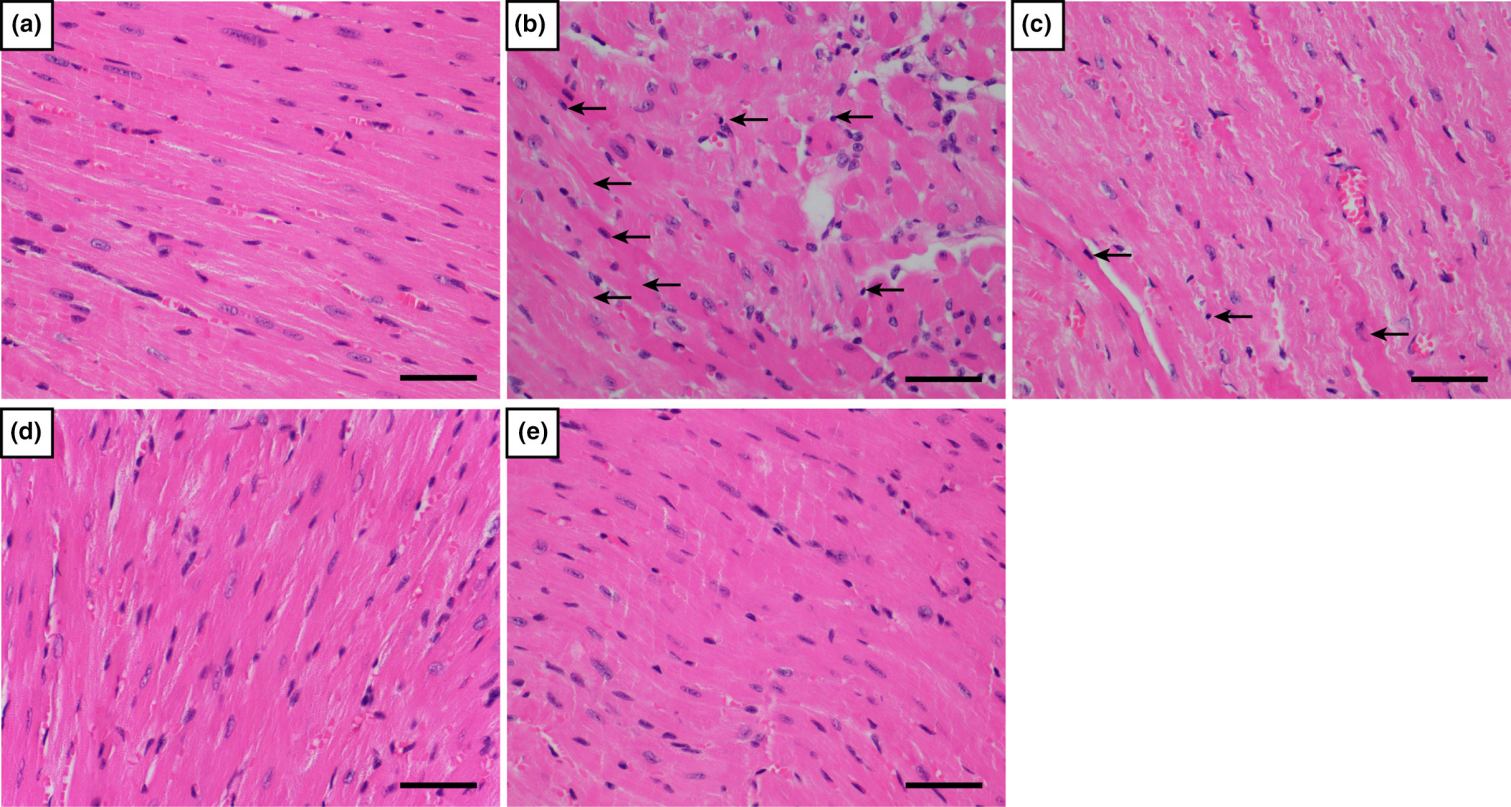
Histopathological changes of the heart between the ISO group and CA treatment groups (scale bar = 50 μm, magnification: 400×). The results of H&E staining shown in (a–e) represent the Cont, ISO, CA‐L, CA‐H, and CA groups, respectively. Arrows indicate cell damage.

H&E staining (Figure 2) showed that in the heart tissue of the Cont group, the myocardium, tissue myocytes, and fibroblasts were arranged in order, with clear nuclei, and regular myocardial striations without atrophy or hypertrophy, indicating normal myocardial structure. Myocardial cells in the ISO group showed obvious inflammatory cell infiltration, cell necrosis, and obvious disorder of the myocardial cells. The degree of myocardial tissue injury was improved in the CA treatment group.

#### 3.1.2 Masson's trichrome staining

**FIGURE 3:**
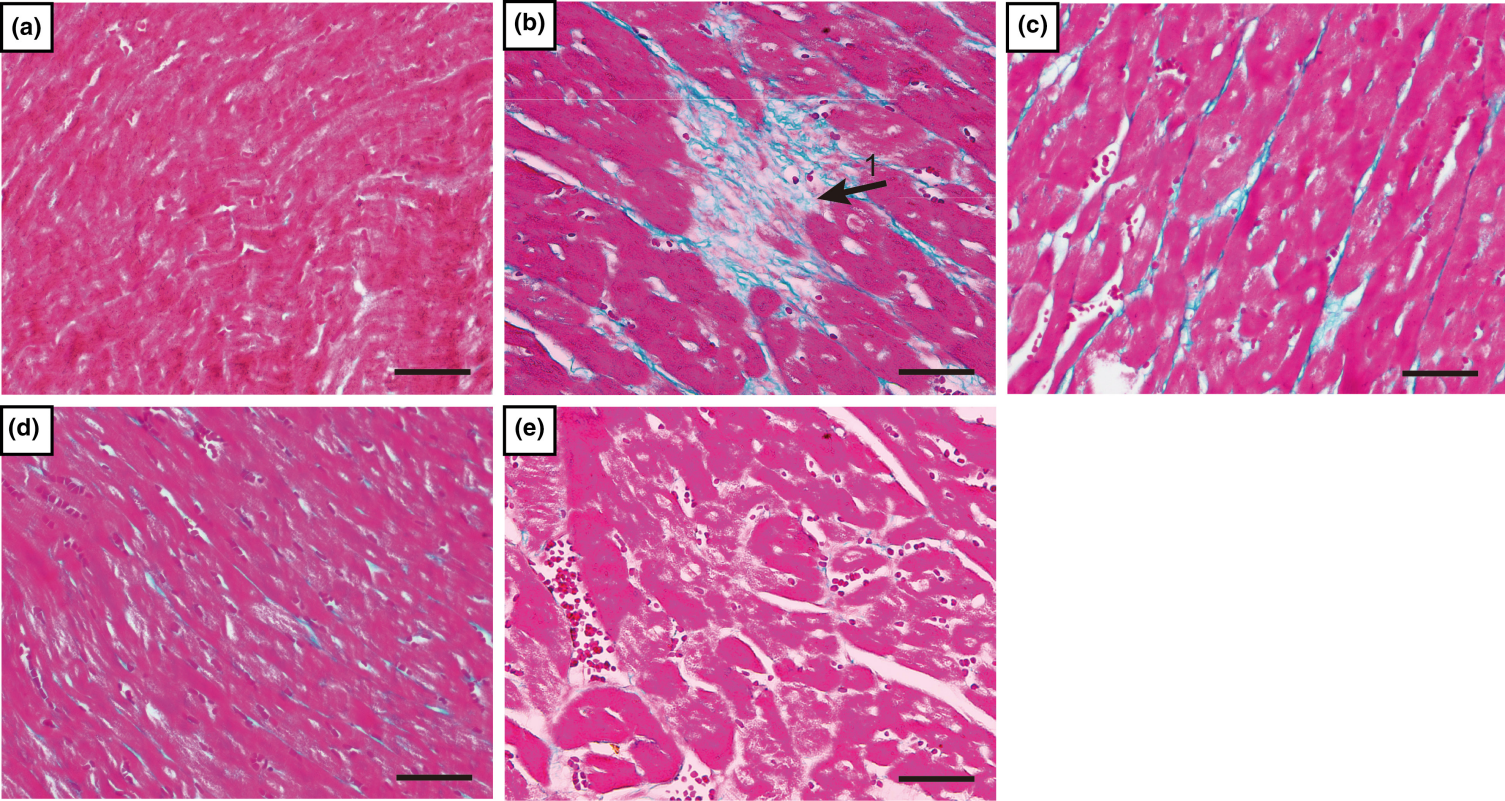
Masson's trichrome staining of the heart showing changes between the ISO group and CA treatment groups (scale bar = 50 μm, magnification: 400×). Arrow 1 shows the deposition of collagen fibers in the space between cardiomyocytes.

Masson's trichrome staining results showed that injection of ISO could lead to myocardial collagen deposition (Figure 3), while CA could relieve MF caused by ISO and relieve myocardial collagen deposition.

### 3.2 Effects of CA on ISO‐induced changes in myocardial enzymes

**FIGURE 4:**
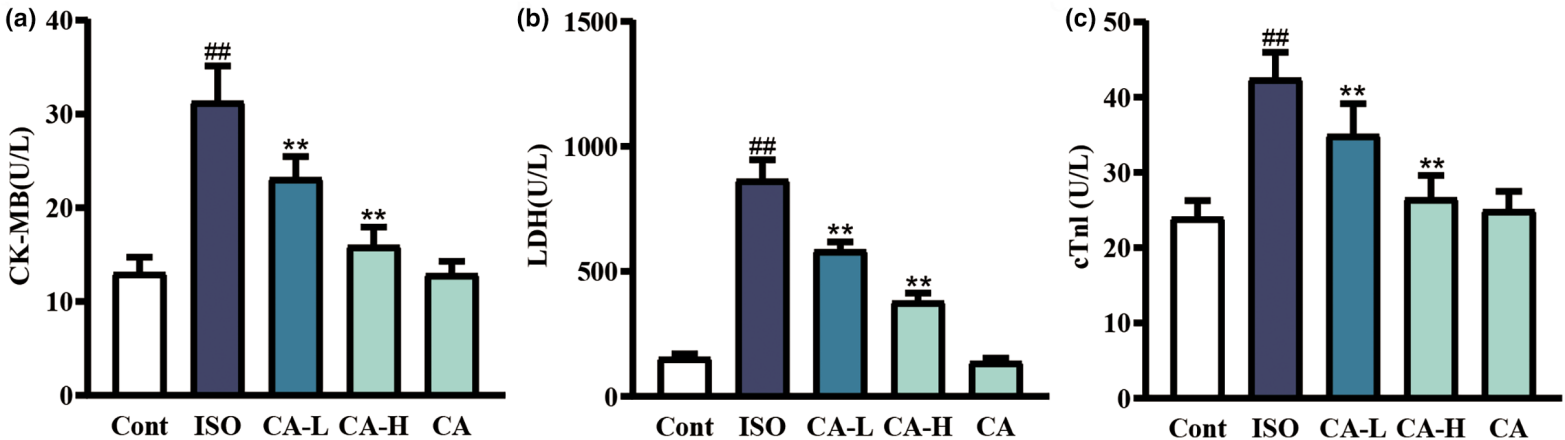
Changes in CK‐MB (a), LDH (b), and cTnI (c) expression are shown between the ISO group and CA treatment groups. Values are expressed as mean ± SD (*n* = 6; compared with the Cont group, ^##^ *p* < .01; compared with the ISO group, **p < .01).

In order to observe the effect of CA on ISO‐induced myocardial enzymes, mouse serum was detected using serum detection kits (Figure 4). The results indicated that injection of ISO increased the levels of CK‐MB, LDH, and cTnI compared to the Cont group (*p* < .01). CA can ameliorate ISO‐induced elevation of serum markers.

### 3.3 Effect of CA on ISO‐induced changes in antioxidant activity

**FIGURE 5:**
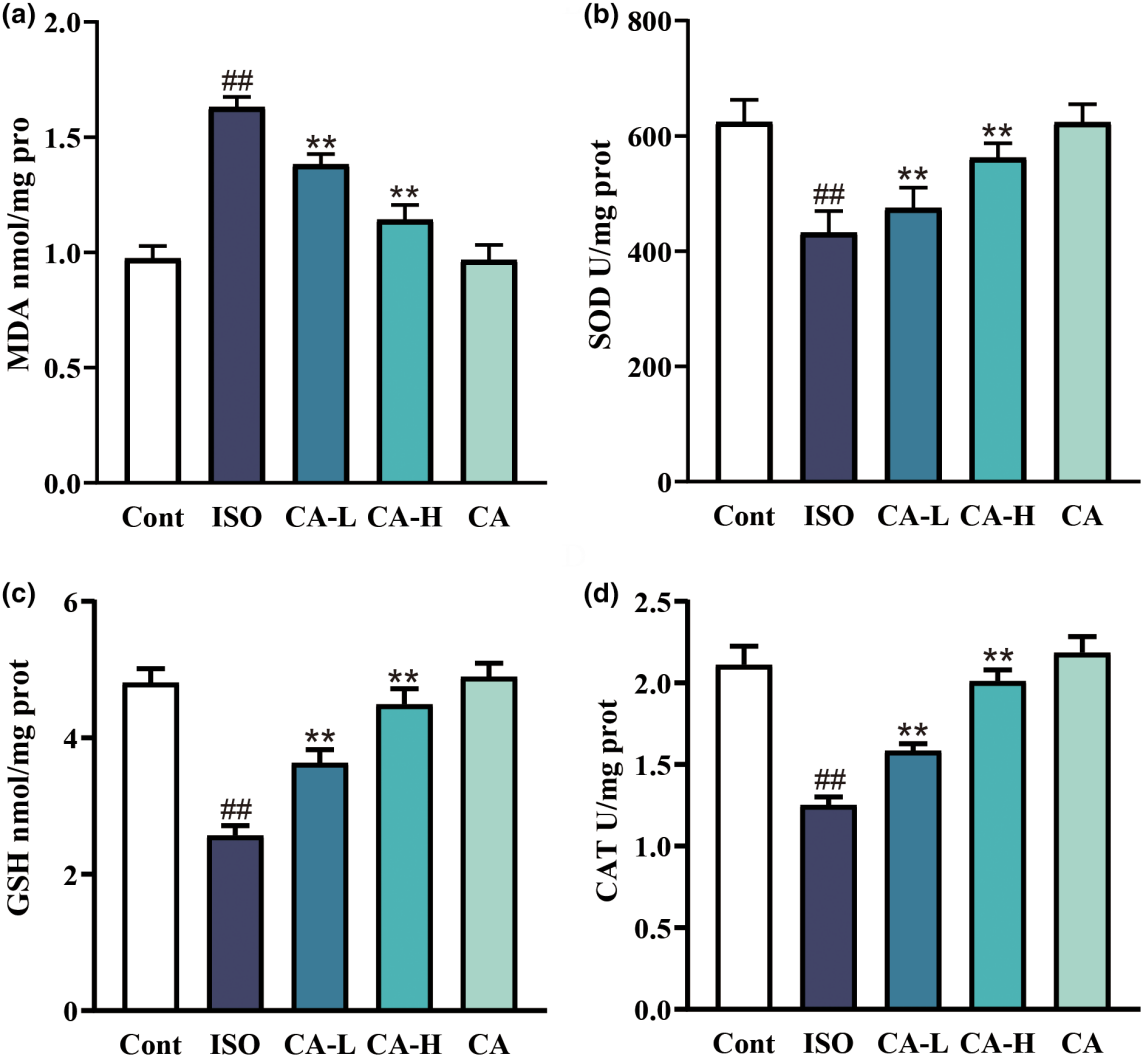
Changes in MDA (a), SOD (b), GSH (c), and CAT (d) expression are shown between the ISO group and CA treatment groups. Values are expressed as mean ± SD (*n* = 6; compared with the Cont group, ^##^ *p* < .01; compared with the ISO group, **p < .01).

To verify that CA alleviates oxidative stress damage in MF, we examined several oxidative stress‐related indicators. As shown in Figure 5, the MDA level significantly decreased in the ISO group but not in the Cont group, while SOD, CAT, and GSH levels significantly increased. Compared with the ISO group, the MDA levels in the CA treatment groups significantly increased, while SOD, CAT, and GSH levels significantly decreased.

### 3.4 Effect of CA on ISO‐induced changes in myocardial mitochondrion ultrastructure

**FIGURE 6:**
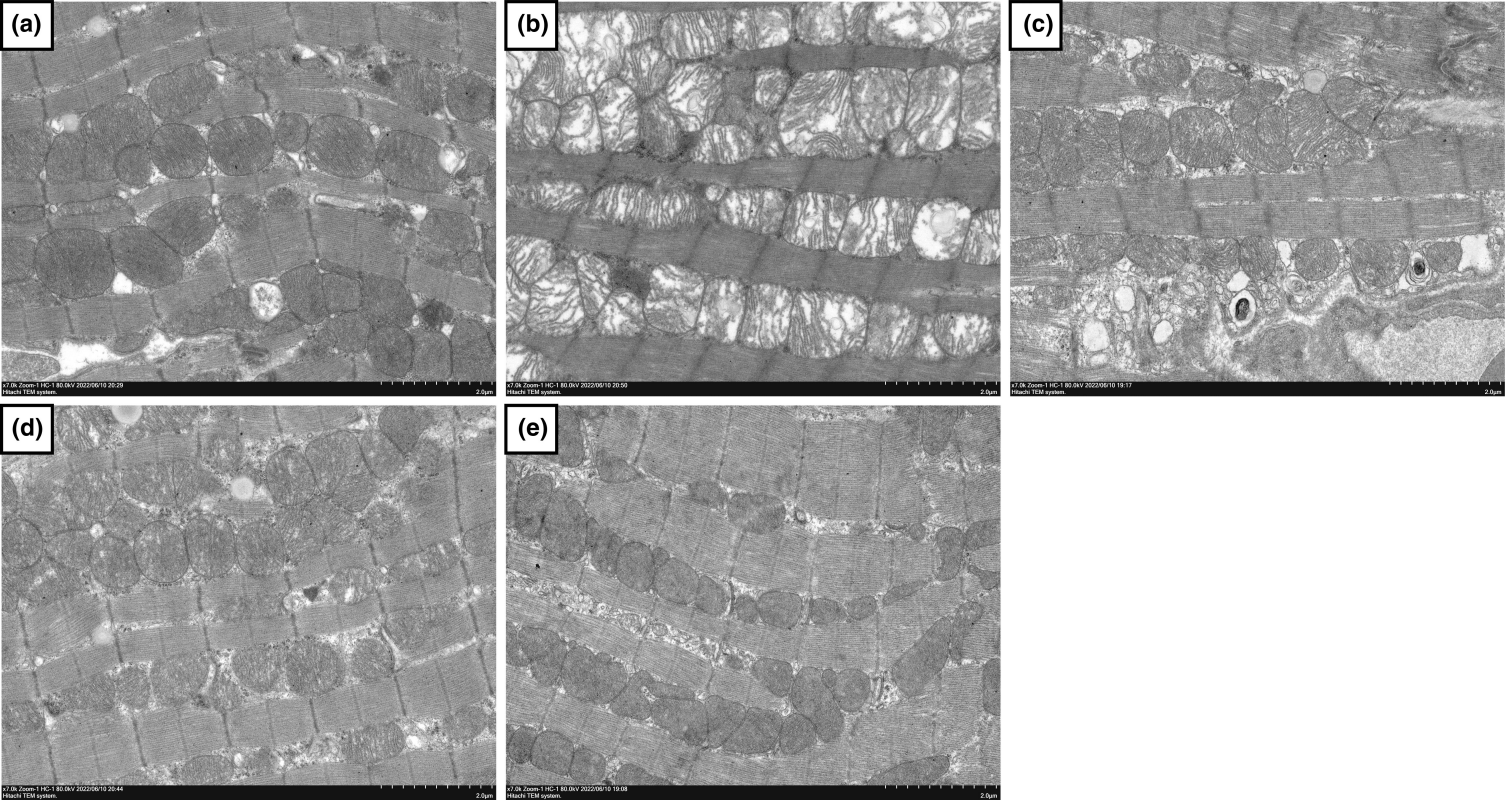
Changes in myocardial mitochondrion ultrastructure between the ISO group and CA treatment groups (scale bar = 2.0 μm, magnification: 7000×). (a–e) Cont, ISO, CA‐L, CA‐H, and CA groups, respectively.

The ultrastructural changes of myocardial mitochondria were observed by TEM (Figure 6). In the ISO group, most mitochondria were damaged, including enlargement of the cristae space; shortening of the cristae, breakage, or disappearance (mitochondria were cystic); enlargement of the myofibrillar space (cardiomyocytes were slightly edematous); and disruption, lysis, and disappearance of some myofilaments. The degree of damage to the CA‐L and CA‐H mitochondria was alleviated, the cristae space was reduced, and the arrangement of myofibrils was regular, especially in the CA‐H group.

### 3.5 Effect of CA on ISO‐induced changes in ROS expression

**FIGURE 7:**
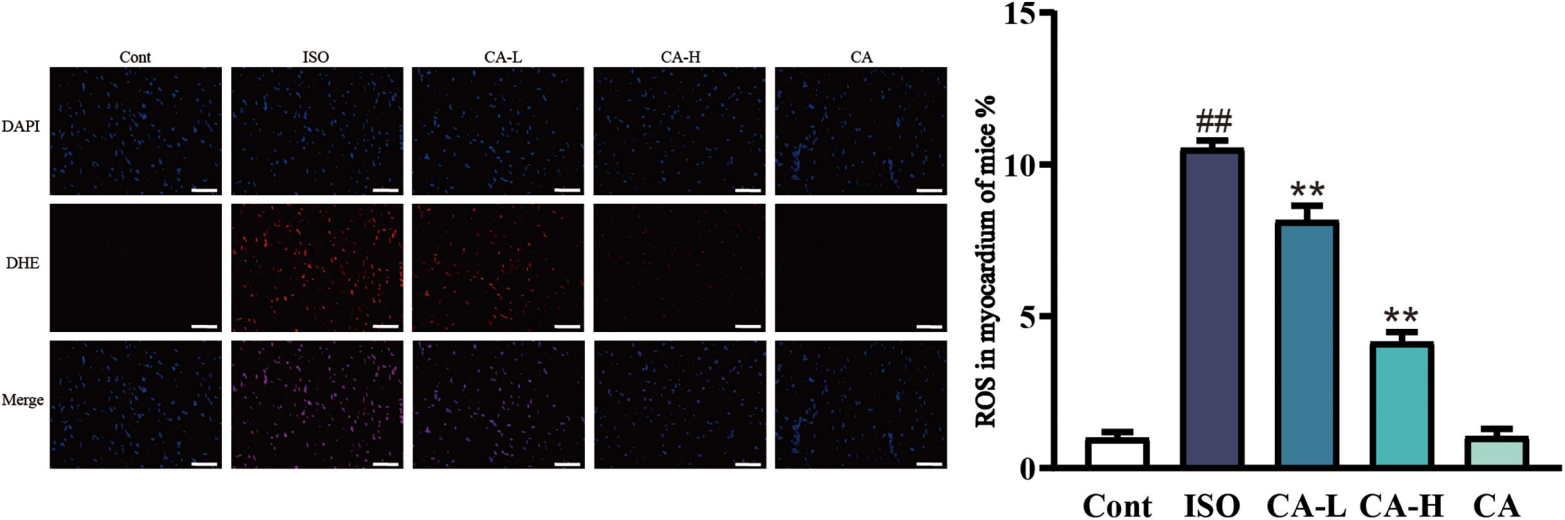
Changes in ROS expression between the ISO group and CA treatment groups. Scale bar = 50 μm, magnification: 400×. (n=3, ^##^ *p* < .01, compared to the Cont group; **p < .01, compared to the ISO group).

In Figure 7, the fluorescence results show that the ROS fluorescence intensity of the ISO group was significantly enhanced compared to the Cont group. The CA‐L and CA‐H treatments effectively restrained ROS overexpression induced by ISO.

### 3.6 Effect of CA on ISO‐induced changes in the expression of inflammatory cytokines

**FIGURE 8:**
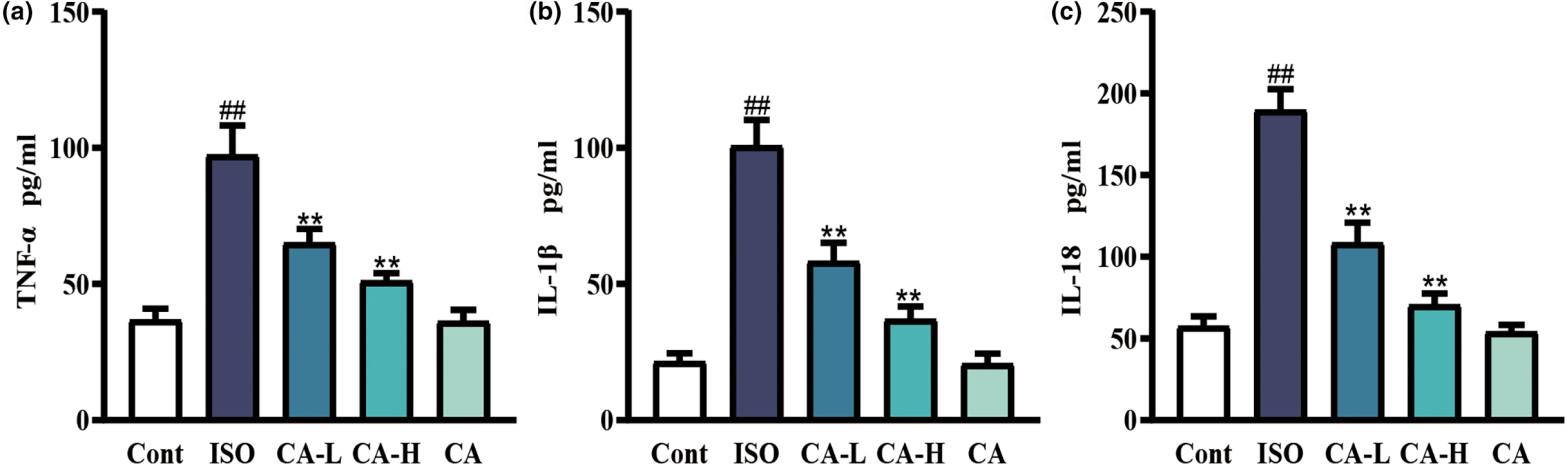
Changes in TNF‐α (a), IL‐1β (b), and IL‐18 (c) between the ISO group and CA treatment groups. Results are expressed as mean ± SD (*n* = 6; ^##^ *p* < .01, compared to the Cont group; **p < .01, compared to the ISO group).

The expression of proinflammatory factors was detected using ELISA kit (Figure 8). ELISA results showed that the expression of TNF‐α, IL‐18, and IL‐1β increased in the ISO group (*p* < .01), but not in the Cont group. The levels of TNF‐α, IL‐18, and IL‐1β in the CA‐L and CA‐H groups were lower than those in the ISO group (*p* < .01).

### 3.7 Effects of CA on ISO‐induced changes in apoptosis

**FIGURE 9:**
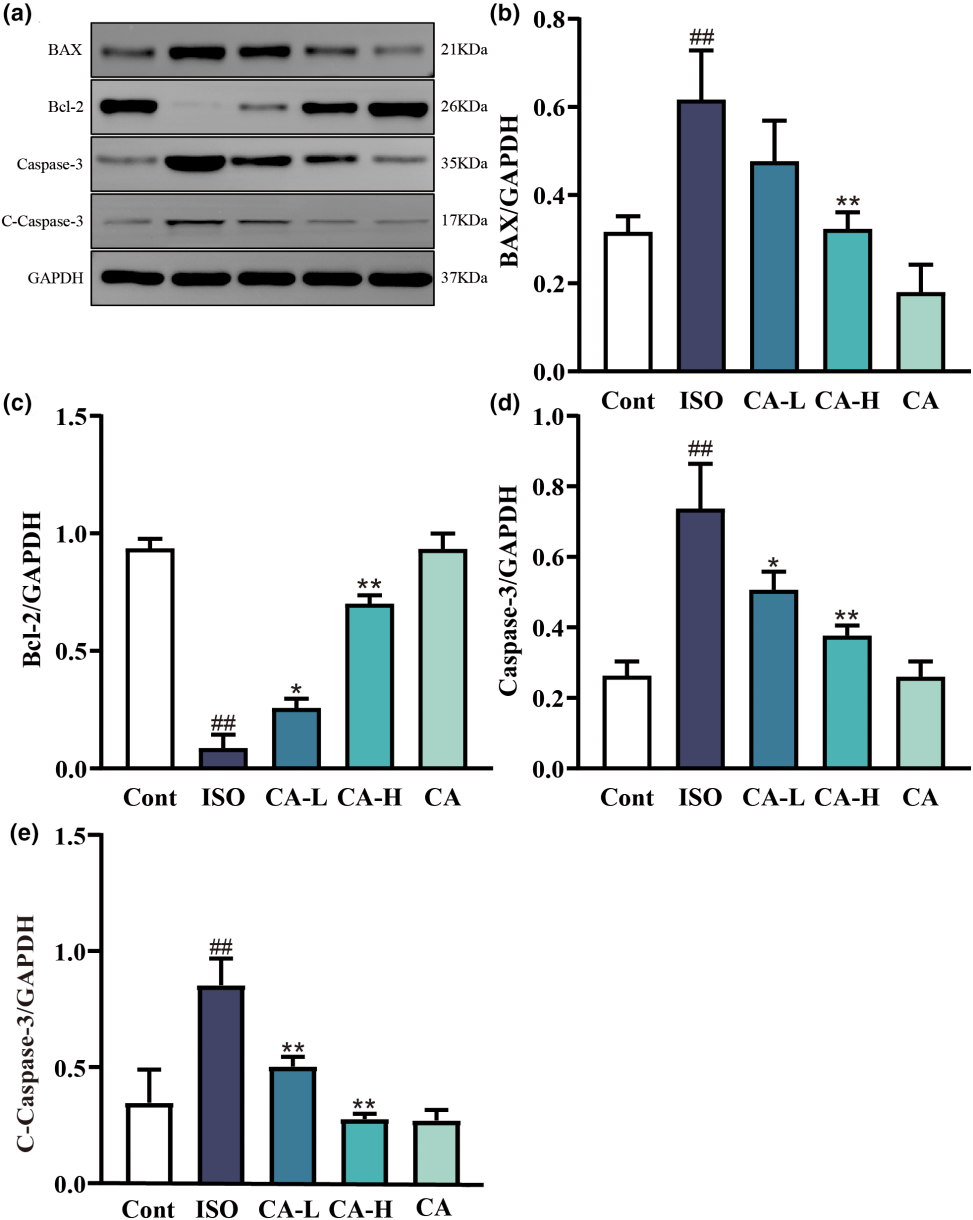
The effects of CA treatment on Bax, Bcl‐2, caspase‐3, and C‐caspase‐3 experessions as showns by western blotting (a). Changes in Bax (b), Bcl‐2 (c), caspase‐3 (d), and C‐caspase‐3 (e) protein expression between the ISO group and CA treatment groups. Results are expressed as mean ± SD (*n* = 3; ^##^ *p* < .01, compared to the Cont group; *p < .05 **p < .01, compared to the ISO group).

We analyzed the expression of apoptosis‐related pathway proteins by western blotting (Figure 9). The results showed that Bcl‐2 expression was downregulated, while the expression levels of Bax, caspase‐3, and cleaved‐caspase‐3 were upregulated in the ISO group but not in the Cont group (*p* < .01). CA improved apoptosis induced by ISO.

### 3.8 Effects of CA on ISO‐induced changes in HK1, NLRP3, and ASC expression

**FIGURE 10:**
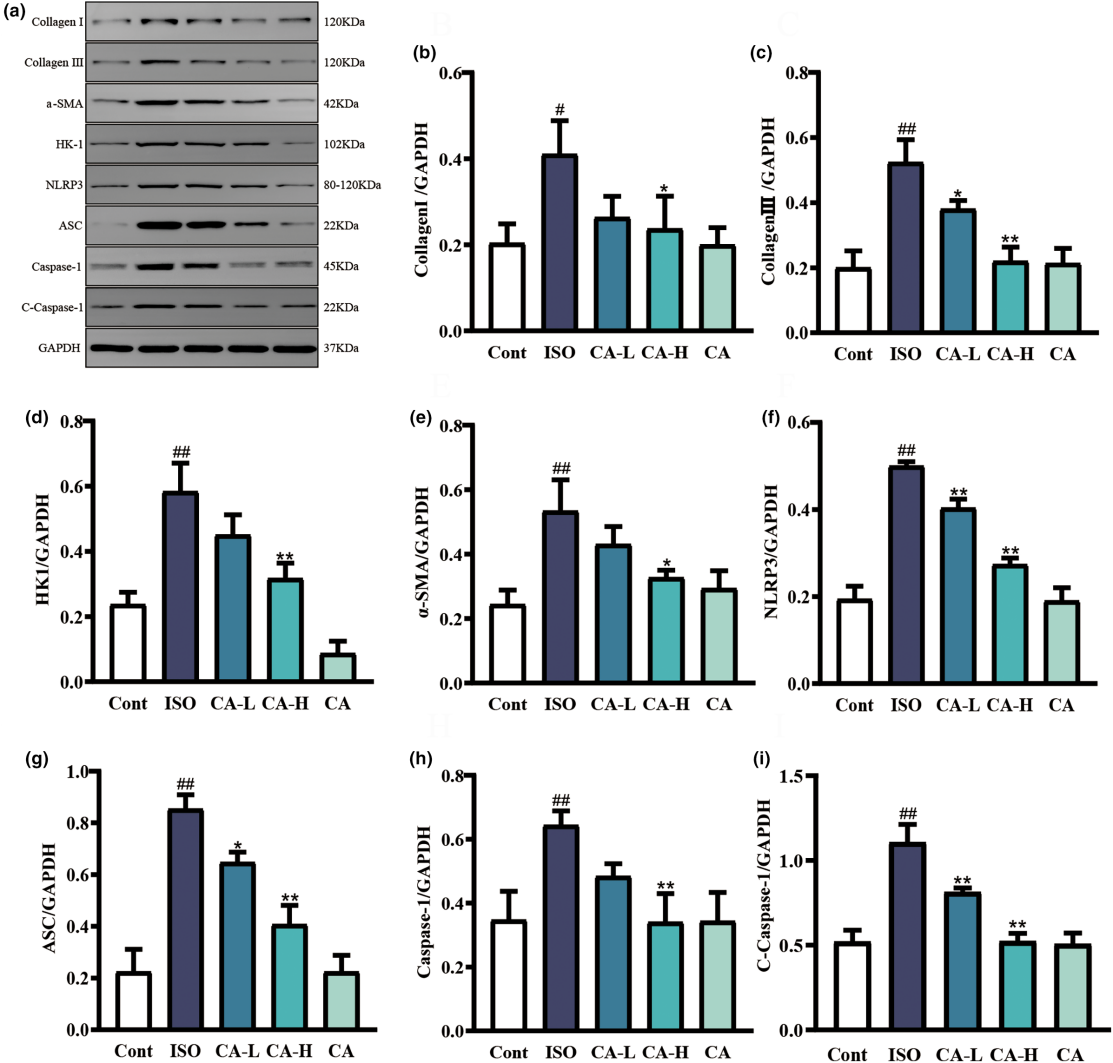
The effects of CA treatment on collagen I, collagen III, HK1, α‐SMA, NLRP3, ASC, caspase‐1, and C‐caspase‐1 expressions as showns by western blotting (a). Changes in collagen I (b), collagen III (c), HK1 (d), α‐SMA (e), NLRP3 (f), ASC (g), caspase‐1 (h), and C‐caspase‐1 (I)  protein expression between the ISO group and CA treatment groups. Results are expressed as mean ± SD (*n* = 3; ^#^ *p* < .05 and ^##^ *p* < .01, compared to the Cont group; *p < .05, **p < .01 compared to the ISO group).

**FIGURE 11:**
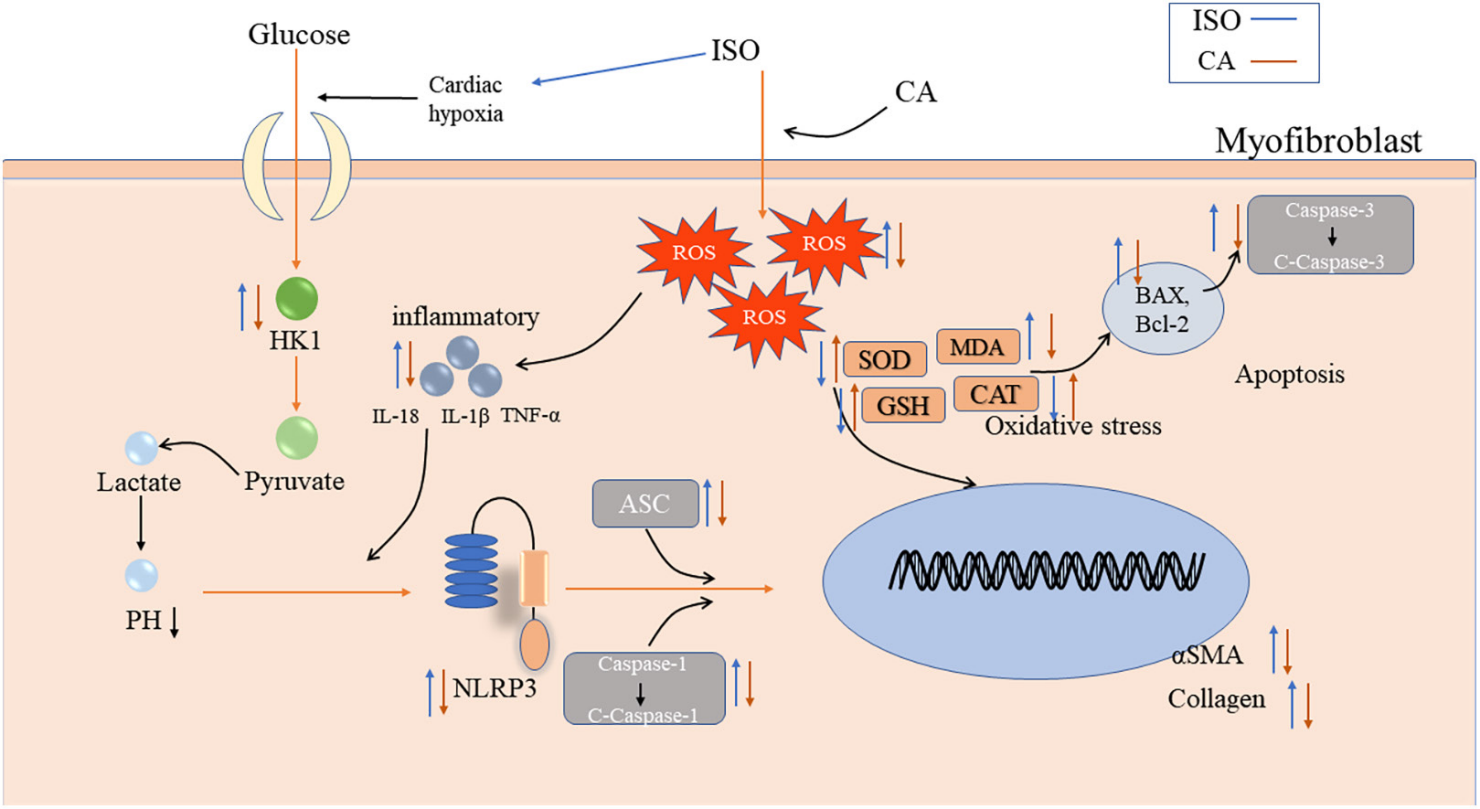
Mechanism for the protective effects of CA against ISO‐induced myocardial fibrosis.

The results showed that the NLRP3 signaling pathway can be activated by analyzing ISO injection. CA improved the degree of ISO‐induced fibrosis (Figure 10). As shown in Figure 11, compared with the Cont group, the levels of pathway‐related proteins HK1, NLRP3 ASC, caspase‐1, and cleaved‐caspase‐1 were significantly increased during injection ISO (*p* < .01). CA improved this phenomenon.

## 4 DISCUSSION

MF can lead to refractory heart failure, malignant arrhythmia, and myocardial infarction (Wang et al., 2019). However, there is no specific drug for the treatment of MF. Therefore, it is necessary to find effective therapeutic targets and drugs for MF. ISO, a β‐adrenergic receptor agonist synthesized in this study, has been widely used to induce cardiac fibrosis in animal models (Han et al., 2020). After ISO‐induced MF, there was excessive proliferation of myocardial fibroblasts, and excessive expression and disordered deposition of ECM proteins. These together led to oxidative stress, inflammation, apoptosis, MF, and cardiac dysfunction (Liang et al., 2020). It has been recently reported that CA is a compound with antioxidant and anti‐inflammatory activities, and that CA has a strong cardiovascular protective effect (Zhu et al., 2017). This study confirmed that CA can improve MF induced by injection of ISO. CA improved the cardiac function of ISO‐induced MF by attenuating ISO‐induced pathological changes and inhibiting collagen deposition in MF (Amran et al., 2015). CA inhibited MF by reducing inflammation, apoptosis, and oxidative stress. CA was also shown to inhibit NLRP3 activation by inhibiting HK1, and then subsequently inhibiting NLRP3‐induced MF caused by the inflammasome signaling pathway.

In order to determine the degree of myocardial injury, cardiac tissue was pathologically observed in this study. Two weeks after CA injection, HE staining and Masson's trichrome staining showed that the ISO group showed dramatic pathological changes compared to the other groups, including apoptotic cells, inflammatory cells, myocardial necrosis (Figure 2), and myocardial interstitial collagen deposition (Figure 3). In the CA treatment group, the results showed that CA can improve ISO‐induced changes in cardiac function. Therefore, we decided to verify the protective effect of CA on the heart by establishing an ISO‐induced MF mouse model. CK‐MB, LDH, and cTnI, as markers of myocardial injury, can observe the degree of heart injury (Amran et al., 2015; Lu et al., 2018). The results (Figure 4) showed that CA improved cardiac serum changes induced by ISO injection. It is suggested that CA can protect the integrity of the myocardial cell membrane by playing an antioxidant role.

The main event occurring in MF is the activation and differentiation of cardiac fibroblasts into myofibroblasts. Myofibroblasts secrete a large amount of ECM protein (Gao, Bo, et al., 2019; Gao, Shi, et al., 2019; Yun et al., 2021); α‐SMA expression is a hallmark of mature myoblasts (Shinde et al., 2017). In addition, cardiac fibroblasts have unique biological functions including increased collagen type I and type III secretion, enhanced α‐SMA expression, and decreased expression of genes encoding ECM degrading enzymes, leading to pathological MF (Gao, Bo, et al., 2019; Gao, Shi, et al., 2019). The western blot results of this study (Figure 10) showed that the expressions of α‐SMA, collagen type I and type III were significantly increased in the ISO model group, indicating successful fibrosis modeling. The CA treatment group inhibited the degree of MF by reversing the expressions of α‐SMA, collagen type I and type III. CA partially reduced MF by inhibiting collagen expression.

The overexpression of ROS is closely related to various stimulus responses. The mass production of ROS changes the structure of DNA in the body, which leads to the changes of proteins and lipids, induces several stress‐induced transcription factors, and is related to anti‐inflammatory response (Priya Dharshini et al., 2020). Previous studies have shown that ROS induced the onset of fibrosis by secreting a variety of profibrotic factors (Xue et al., 2021). Another study showed that CA treatment inhibited the generation of ROS (Lis et al., 2019). In this study, as shown in Figure 7, ROS expression abnormally increased in ISO‐induced MF, while ROS overexpression was inhibited in the CA treatment group. Oxidative stress and ROS act against lipids, proteins, and nucleic acids (Liang et al., 2022). Therefore, we found (Figure 5) that the expression of MDA significantly increased in the ISO group, while the expression of SOD, CAT, and GSH decreased, indicating that oxidative stress damage was aggravated. In the CA treatment group, the expression of MDA decreased, the expression of SOD, CAT, and GSH increased, and the oxidative stress injury was significantly reduced. The Bcl‐2 family is the key factor of apoptosis, and Bax is the most representative proapoptotic protein among them (Li et al., 2013). Oxygen depletion occurs in MF, resulting in ROS overexpression, upregulation of Bax, release of cytochrome, cleavage of caspase‐9, and activation of mitochondrial apoptosis pathway to regulate apoptosis. In this study (Figure 9), CA successfully inhibited ISO‐induced increases in Bax, Bcl‐2, and caspase‐3. The results indicated that CA inhibited apoptosis of cardiomyocytes.

Interstitial fibrosis is part of an inflammatory process that causes fibroblasts present in a healthy heart to differentiate into myofibroblasts and mesenchymal cells during disease, further leading to fibrosis. Fibroblast activation is induced by cellular inflammation and by cytokines and growth factors secreted by cardiomyocytes (Khalil et al., 2017; Nagaraju et al., 2019). Previous studies have shown that CA has a strong anti‐inflammatory effect and can induce inflammasome activation by inhibiting lipopolysaccharide‐ATP secretion of proinflammatory cytokines, such as IL‐18 and IL‐1β (Yansong Xue et al., 2017), which are key factors of cardiac inflammation. Literature studies have found that inhibition of NLRP3 expression inhibited the expression of IL‐1β and IL‐18, which further reduced inflammation. The inflammasome of NLRP3 plays a key role in recognizing danger signals and inducing sterile inflammatory responses in myocardial infarction (Mezzaroma et al., 2011; Sandanger et al., 2013). This facilitates the processing of pro‐IL‐18 and pro‐IL‐1β into biologically active mature forms (Latz et al., 2013). In addition, by inhibiting the expression of NLRP3, the inflammatory response after myocardial infarction can be reduced, which can protect myocardial function and improve myocardial remodeling. In our study, we found that the NLRP3 inflammasome was activated in ISO‐induced MF, protein expression levels of ASC, caspase‐1, cleaved caspase‐1 were increased, and CA inhibited the activation of the NLRP3 inflammasome. The protein expression of caspase‐1 and cleaved caspase‐1 was inhibited. It has been reported that inhibiting the expression of NLRP3 can inhibit the expression of IL‐1β and IL‐18, so as to further reduce the inflammatory response. This conclusion is consistent with the conclusion of this study, proving that CA can inhibit the activation of NLRP3 bodies, reduce the inflammatory response, and thus inhibit the degree of fibrosis. It is known that glycolysis is involved in MF after myocardial infarction and NLRP3 inflammasome is activated by glycolytic inflammation, and HK1 is the first step of glycolysis. This study hypothesized that HK1 is involved in the activation of NLRP3 inflammasome. In MF caused by ISO, the damage of myocardial tissue causes glycolysis to supply energy, and the expression of HK1 protein increases. Activation of the NLRP3 inflammasomes resulted in increased protein expression levels of ASC, caspase‐1, cleaved caspase‐1, and increased expression of IL‐18 and IL‐1β. CA inhibited the increase of HK1 by playing an antioxidant and anti‐inflammatory role, and then regulated the growth of NLRP3 inflammasome, thereby reversing the protein expression in the HK1/NLRP3 pathway. Inhibiting the degree of fibrosis has a protective effect on myocardial injury.

## 5 CONCLUSION

We demonstrated that CA had a significant protective effect against ISO‐induced MF. The cardioprotective effects of CA arise through anti‐inflammatory, antiapoptotic, and antioxidant mechanisms associated with the regulation of the HK1/NLRP3 inflammasome pathway. Based on these findings, CA may be a promising drug for future clinical applications.

## AUTHOR CONTRIBUTIONS

**Donglai Ma:** Writing – review and editing (equal). **Xizhen Cheng:** Writing – original draft (lead). **Yuling Zhang:** Visualization (equal). **Haochuan Guo:** Investigation (supporting). **Xinnong Li:** Investigation (supporting). **Yanan Wang:** Investigation (supporting). **Hongfang Wang:** Methodology (supporting). **Yongxing Song:** Software (supporting).

## FUNDING INFORMATION

This work was supported by the Natural Science Foundation of Hebei Province of China (No. H2022423297) and the S&T Program of Hebei of China (No. CXZZSS2023107).

## CONFLICT OF INTEREST STATEMENT

The authors report no conflicts of interest.

## ETHICS STATEMENT

The experimental procedures and protocols were approved by the Committee on the Ethical Use of Animals of Hebei University of Chinese Medicine (DWLL2021106).

## Data Availability

The data that support the findings of this study are available from the corresponding author upon reasonable request.
